# Invasion Risk and Potential Impact of Alien Freshwater Fishes on Native Counterparts in Klang Valley, Malaysia

**DOI:** 10.3390/ani11113152

**Published:** 2021-11-04

**Authors:** Abdulwakil Olawale Saba, Ahmad Ismail, Syaizwan Zahmir Zulkifli, Intan Faraha A. Ghani, Muhammad Rasul Abdullah Halim, Musa Adamu Ibrahim, Aqilah Mukhtar, Azharuddin Abd Aziz, Noor Azrizal Abdul Wahid, Mohammad Noor Azmai Amal

**Affiliations:** 1 Department of Biology, Faculty of Science, University Putra Malaysia, Serdang 43400, Selangor, Malaysia; sabaola@gmail.com (A.O.S.); aismail@upm.edu.my (A.I.); syaizwan@upm.edu.my (S.Z.Z.); moosaad8@gmail.com (M.A.I.); aqilahmukhtar90@gmail.com (A.M.); 2 School of Agriculture, Epe Campus, Lagos State University, Lagos 106101, Nigeria; 3 Department of Science and Biotechnology, Faculty of Engineering and Life Sciences, Bestari Jaya Campus, University Selangor, Bestari Jaya 45600, Selangor, Malaysia; intanfaraha@unisel.edu.my; 4 School of Biological Sciences, University Sains Malaysia, Gelugor 11800, Penang, Malaysia; mrasul87@gmail.com; 5 Department of Biological Sciences, University of Maiduguri, Maiduguri P.M.B. 1069, Nigeria; 6 Department of Chemistry Malaysia, Ministry of Science, Technology and Innovation, Jalan Sultan, Petaling Jaya 46661, Selangor, Malaysia; azharuddin@kimia.gov.my; 7 Institute of Advanced Studies, University of Malaya, Kuala Lumpur 50603, Malaysia; azrizal_wahid@yahoo.com

**Keywords:** community structures, invasive fishes, anthropogenic factors, potential invasiveness screening, diet overlap, stable isotope analysis

## Abstract

**Simple Summary:**

The mechanisms on how alien species naturally affect the native species in the real aquatic environment are infrequently studied. This study explores the potential effects of alien fishes on the native fish community, well-being, and trophic preferences in selected rivers of Klang Valley, Malaysia. We found that alien fishes benefited from the impacts of anthropogenic activities in their surrounding habitats, while their plasticity in feeding habits might help them to invade, survive, and dominate. This study revealed the natural mechanisms on the establishment of alien fish species and their potential ecological impacts on native fishes in the rivers of Klang Valley, Malaysia.

**Abstract:**

This study explores the potential effects of alien fishes on the native fish community, well-being, and tropic preferences in selected rivers of Klang Valley, Malaysia. Following the Aquatic Species Invasiveness Screening Kit assessment, most of the alien fishes (80%) are invasive. The alien species occurrences correlated positively (*p* < 0.05) with poor water quality, such as rivers with high ammonia-nitrogen and nitrite, but negatively with phosphate and dissolved oxygen. Anthropogenic characteristics, such as rivers with high pollution levels and ease of accessibility to the fish habitat, are mainly associated positively (*p* < 0.05) with the occurrences of alien fish species. In general, the results of fish stomach contents analyses and their associated indices, together with stable carbon and nitrogen isotopes, revealed domination by alien fishes or diet overlaps between both alien and native fish species. This finding indicates that alien fishes benefited from the impacts of the anthropogenic activities in their surrounding habitats, while their plasticity in feeding habits might help them to invade, survive, and dominate in the rivers of Klang Valley, Malaysia.

## 1. Introduction

Alien fishes threaten native biodiversity and health through food and space competition, predation, hybridization, habitat and trophic modifications, and the introduction of diseases [1,2]. They are usually imported for beneficial purposes, including the need to boost a country’s fish production, the use of these fishes as ornaments, for sports, and biological control of unwanted species [3,4]. Unfortunately, when these fishes get intentionally or accidentally introduced into the local waters, and the negative impacts get to the peak leading to domination of the environment, alien fishes become invasive [5,6]. Aquatic bioinvasions have generated environmental issues globally, affecting fresh, marine, and brackish water ecosystems worldwide. Moreover, fishes represent an important aquatic group that has witnessed broad introduction and translocation basically to enhance, restore, and re-establish fisheries resources [7,8]. Therefore, invasive species are regarded as one of the most critical threats to freshwater ecosystems functioning and health [9].

Evaluating the potential and existing impacts of alien fish species is quintessential [10]. Information from such assessments may facilitate the understanding of the ecological and socio-economic implications arising from their introduction, establishment, spread, and subsequent invasion [11]. In so doing, the impact assessment of alien fishes on native fish species involves studying the risk of non-native fish invasion, occurrence, diversity, well-being, and food preferences of both alien and native fishes [12,13]. The decline in native fish occurrences, diversity, the corresponding increase in alien fish abundance, the comparatively better growth condition of alien fishes, the predation on natives, and competition of aliens with natives for food and space have been used to measure the impacts generated by alien fishes [14,15]. Moreover, to detect and quantify predation and food competition between alien fishes and their native counterparts, analyses of their diets have also been carried out [16]. Moreover, diet overlap has been assessed by paired diet comparisons between native and alien species [16,17,18]. Despite its value in evaluating the trophic relationship of fish species, stomach contents analysis can best consider the diet in a short-term period. Moreover, it depends on the skill and expertise of the assessors [19]. To complement the information resulting from fish stomach contents analyses, stable isotope analysis, primarily carbon (δ^13^C) and nitrogen (δ^15^N) has been widely used [20,21,22,23]. Thus, stable isotope analysis has helped to reveal the trophic interactions between native and alien fish species in conjunction with stomach contents analysis [24].

In Malaysia, alien fishes such as tilapia (*Oreochromis* sp.), sailfin catfishes (*Pterygoplichthys pardalis* and *Pterygoplichthys disjunctivus*), and guppy (*Poecilia reticulata*) have been reportedly introduced both intentionally and inadvertently for different purposes [3,25,26]. Therefore, alien fishes are present and, in some cases, have established breeding populations in the inland waters of this country [27]. The presence and establishment of certain alien fish species in the local water bodies within Klang Valley, Malaysia, have been reported [28,29,30,31]. However, the mechanisms on how the alien species affect the native species in the real aquatic environment are limited. Due to the shortage of scientific information regarding the potential impacts of alien fishes in Malaysia, the introduction of other alien species may continue, which may translate to huge ecological and economic costs. Thus, the objectives of this study are: (1) to determine the fish community structures (including native and alien fishes) and relate their occurrences with environmental characteristics in selected rivers within Klang Valley, Malaysia; (2) to assess the invasion risks of alien fish species in selected rivers of Klang Valley, Malaysia, using the Aquatic Species Invasiveness Screening Kit (AS-ISK); and (3) to identify the stomach contents, trophic level, and diet overlap of alien and native fish species from stomach contents analysis, and compare the stable carbon (δ^13^C) and nitrogen (δ^15^N) signatures of alien and native fishes. 

## 2. Materials and Methods

### 2.1. Study Area

Peninsular Malaysia has a warm and humid tropical climate, and it lies between 1° and 7° north and between 99° and 105° east, with an area of 132,000 km^2^. It experiences a uniform temperature all year round, and rainfall usually occurs in two seasons: the northeast monsoon from November to March and the southwest monsoon from May to September [32,33]. In Peninsular Malaysia, Klang Valley is the most densely populated area covering seven central districts, such as the Federal Territory of Kuala Lumpur, Gombak, Hulu Langat, Kuala Langat, Sepang, Klang, and Petaling. It is positioned at the center of the western coast of Peninsular Malaysia and is approximately 2832 km^2^ in area [34]. 

**Figure 1:**
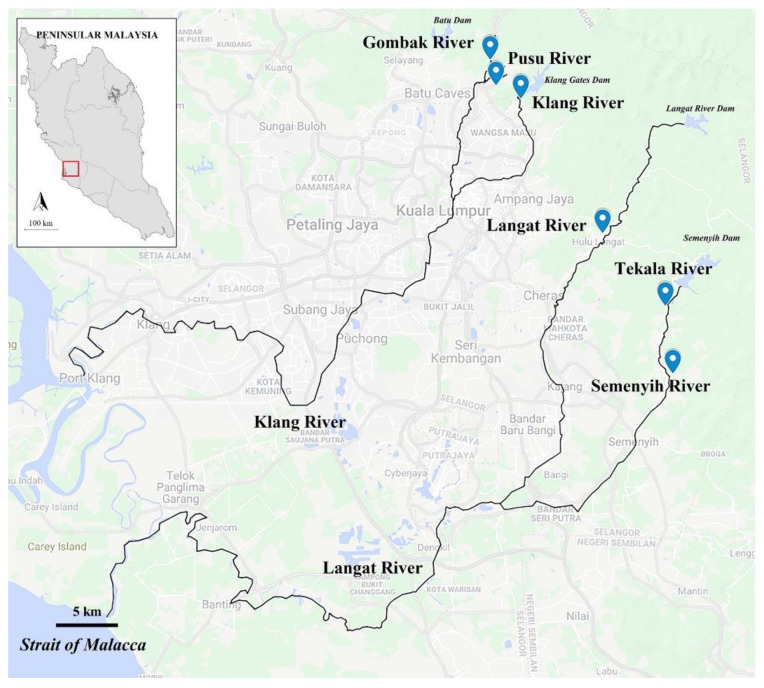
Map of Peninsular Malaysia showing sampling sites within the Klang Valley, Malaysia. Black lines indicate the rivers. Source: Adapted from Google Map at www.google.com.my/maps/ on 1 June 2021.

Six rivers within Klang Valley were selected for this study (Figure 1). The sampling took place from January 2020 to March 2020. Despite having all sampling sites within the Klang Valley where some of them experience some anthropogenic influences, some rivers, such as the Pusu, Langat, Gombak, and Klang Rivers, were selected due to documented and anecdotal reports on the presence of certain alien fish species. In contrast, others, such as the Tekala and Semenyih Rivers were chosen without prior information about alien fish species. In each river, at least a distance averaging 500 m was covered for fish and water sample collection. The environmental conditions, characteristics, and coordinates of each river are described in Supplementary Material Table S1.

### 2.2. Fish Sampling

A cast net of 150 cm long and 305 cm in diameter with a mesh size of 2 cm and scoop nets ranging from 2 mm to 3 mm mesh sizes were used for fish sampling. To ensure the uniformity of sampling efforts across sites, we used the same gear for a similar duration of four effort hours (10.00 a.m. to 2.00 p.m.) by four individuals. Along each 500 m stretch, fish samples were collected at different points based on eye estimation for a similar duration and effort across the rivers. After collecting the fish samples, they were identified, enumerated, and distinguished as either native or alien by using a combination of keys from Kottelat et al. [35] and Zakaria-Ismail et al. [36]. The samples were also measured for standard length (SL) and total length (TL) to the nearest 0.1 mm, and body weight to the nearest 0.1 g of an individual fish specimen using a metal ruler and an electronic weighing scale (Camry, Guandong, China). The fishes used for further analysis were sedated with overdose tricaine methanesulphonate (MS-222 at 50 mg/L; Sigma-Aldrich, Kuala Lumpur, Malaysia) and later stored in ice packed styrofoam boxes for onward transportation to the laboratory, where they were immediately dissected, analyzed, or preserved. 

The fish were sampled, handled, and sacrificed according to the methods approved by the Institutional Animal Care and Use Committee, Universiti Putra Malaysia. All procedures were carried out following relevant guidelines and regulations. No permit was required to conduct the present study, as none of the sampled fish species was considered endangered and protected by the government of Malaysia.

### 2.3. Water Quality Parameters and the Surrounding Anthropogenic Characteristics

Water quality parameters of the river water were measured both in situ and ex situ. The in situ measurements of water temperature (°C), dissolved oxygen (DO) (mg/L), conductivity (μS/cm), total dissolved solids (TDS) (mg/L), pH (1–14), and salinity (ppt) were done using the YSI Pro Plus Handheld Multiparameter water quality meter (YSI, Yellow Springs, OH, USA). Water depth (cm) was measured using a meter ruler, with all measurements done in triplicates. In contrast, the ex situ measurements were done in less than 24 h after collecting the water samples using sterilized 500 mL polyethylene bottles. The ex situ measurement of parameters, such as turbidity (NTU), ammonia-nitrogen (NH_3_-N) (mg/L), nitrite (NO_2_^−^) (mg/L), nitrate (NO_3_^−^) (mg/L), and phosphate (PO_4_^3−^) (mg/L), were done at the laboratory using the HACH multiparameter portable colorimeter (HACH Company, Loveland, CO, USA).

The anthropogenic characteristics of the sampling sites included; (1) accessibility (how easy people can enter and explore the waterbody); (2) level of protection (e.g., structure, such as gates, boundary); (3) pollution level (in the form of visible domestic and industrial waste); (4) usage for other purposes (e.g., domestic water source, recreation); and (5) distance from the human settlement, which were scored on a scale of 1 (very low susceptibility) to 5 (very high susceptibility) (Supplementary Material Table S2). Three different individuals carried out the assessment to reduce bias and increase accuracy at all the sampling points. After that, the average score from the three individuals was used to evaluate the relationship of fish occurrences with the scored anthropogenic factors.

### 2.4. Invasiveness Screening

Risk screening was carried out for the five main alien fishes recorded in this study: *Oreochromis niloticus*, *P. pardalis*, *P. disjunctivus*, *P. reticulata*, and *Barbonymus gonionotus*. The screening was achieved using the Aquatic Species Invasiveness Screening Kit (AS-ISK), a freely downloadable tool accessed on 17/05/2021 at www.cefas.co.uk/nns/tools/ [37]. This tool was an outcome of integrating the revised version of the generic screening module of the European Non-Native Species in Aquaculture Risk Analysis Scheme [38] into the Fish Invasiveness Screening Kit v2 [39].

Of the 55 questions in the AS-ISK, the first 49 questions, also referred to as basic risk assessment (BRA), relate to the taxon’s biogeography/historical and biology/ecology aspects. The remaining six questions involve predicting the likely impacts of climatic conditions on the BRA, referred to as the Climate Change Assessment (CCA). Each question in AS-ISK requires the assessor to compulsorily respond, justify, and provide a level of confidence that ranges from 1 (very low) to 4 (very high). The resulting outcome gives a BRA and a BRA+CCA (composite) score ranging from −20.0 to 68.0 and from −32.0 to 80.0, respectively [40].

Similar to one of its parent tools, the FISK v2, AS-ISK scores below 1 indicate that the assessed species will likely not become invasive in the risk assessment (RA) area. Therefore, it is classified as ‘low risk’ [41,42]. On the other hand, scores higher than 1 indicate that the species poses either a ‘medium risk’ or a ‘high risk’ of invasion. Moreover, a calibration process is carried out specifically for the RA area to obtain the threshold value needed to differentiate between medium and high-risk species. An overall confidence factor (CF) was computed based on the assessor’s level of confidence on each response for a given species as ∑ (CLQi)/(4 × 55) (i = 1, …, 55). CLQi is the certainty for question i, 4 is the maximum achievable certainty value (i.e. ‘very certain’), and 55 is the total number of questions in the AS-ISK tool for each species. Hence, CLQi values range from 0.25 (for all 55 questions with a certainty score of 1) to 1 (with a certainty score of 4). Although multiple assessments are preferred, in this study, assessments were done only by the first author, who is knowledgeable of the biology and ecology of fish. The single evaluation was due to the difficulty of getting additional assessments. Moreover, risk screening studies based on a single assessor are not uncommon [40].

Calibration of the AS-ISK tool for Peninsular Malaysia was based on the Receiver Operating Characteristics (ROC) curve analysis using IBM SPSS, ver. 22.0 (IBM Corp., Chicago, IL, USA) [4]. The ROC evaluates the ability of AS-ISK to distinguish between invasive and non-invasive alien fishes assessed for the RA area, and the Area Under the Curve (AUC) values were recorded. After the ROC analysis, an AS-ISK value that maximizes and at the same time minimizes the false positive rate was selected for both BRA and BRA+CCA using Youden’s *J* statistic [43,44].

### 2.5. Fish Community Structures

The diversity of fish species in the sampling sites were measured using the Shannon–Weaver diversity index $(H^{\prime }=-\overset{\infty }{\underset{i=1}{\sum }}pi\ln pi$) [45], evenness using Pielou’s evenness index $(J=H^{\prime }/\ln S\;$) [46], richness using Menhinick’s richness index (Dmn=S/N ) [47] and dominance using Simpson’s dominance index $(C=\sum {\left(\frac{ni}{N}\right)}^{2}$) [48]. Furthermore, a pairwise comparison of diversity among sites was made using Whittaker’s beta diversity index $\beta w=\left(\frac{s}{\alpha \;}\right)-1$ [49]. All analyses were done using the PAST (ver. 3.25) software [50].

### 2.6. Stomach Contents Analysis

The stomachs of randomly selected individuals of each fish species were removed and initially preserved in 10% formalin then 70% ethanol before examining the contents. Stomach contents examination was done under a stereomicroscope using a 0.2 mm deep, 16 by 16 Fuchs Rosenthal counting chamber. Where possible, a minimum of 30 individuals was used per fish species in each sampling site considered. The observation was done in triplicates after diluting the stomach contents with distilled water between 5 and 10 mL based on the size of the entire stomach contents. A pipette was used to place two drops of the diluted sample on the slide. Magnifications ranging from 4× to 40× were used to view the samples under a binocular Olympus CX 21 light microscope (Shinjuku, Tokyo, Japan) [17,51]. The fish stomach contents were identified using relevant identification keys [52,53,54].

### 2.7. Feeding Intensity, Stomach Fullness Index, Frequency of Occurrence, and Volumetric Measurement

The intensity of feeding as a measure of the degree of fullness of each fish stomach was observed and recorded as 0/4 full (empty), 1/4 full, 2/4 full, 3/4 full, and 4/4 full (full) [18]. Afterward, the feeding intensity (FI) was expressed as a percentage as follows: %FI = (the number of guts containing food/ total number of guts) × 100, where FI = feeding intensity [55].

For the stomach fullness index, prior to the examination under the microscope, the fish stomach was blotted to eliminate excess liquid. Further, food items from each stomach were emptied into a petri dish and weighed to the nearest 0.001 g using a Sartorius BP 221S digital scale (Sartorius, Gaithersburg, MD, USA) [56]. As a simple record of stomach contents data, the occurrence percentage of individual food items in the fish stomachs was assessed according to Hyslop et al. [57] as follows: %FO = (number of stomachs containing a prey item/ all non – empty stomachs) × 100, where FO = frequency of occurrence.

For the volumetric measurement, with the aid of the binocular Olympus CX 21 light microscope (Shinjuku, Tokyo, Japan), food items were identified and ranked depending on size and abundance by allotting points [18,55]. These points were summed and converted into corresponding volumes as follows: %V = (number of points allocated to the food component/ total points allocated to subsample) × 100.

### 2.8. Index of Preponderance, Diet Overlap, and Trophic Level

The index of preponderance was applied as follows: $I_{i}=\left(v_{i}\frac{o_{i}}{\sum^{\;}v_{i}o_{i}}\right)\times 100$, where *I_i_* = the index of preponderance, while $v_{i}$ and $o_{i}$ represent the volume and occurrence indices of food item *i*, respectively [58]. For diet overlap, it was assessed using the Morisita-Horn Index (C_H_) in order to measure the potential for competition between alien and native fishes. The C_H_ was determined using the equation as follows: $C_{H}=\;2\sum^{\;}pij\times pik/\sum^{\;}p^{2}\;ij\;\pm \sum^{\;}p^{2}ik$, where C_H_ = diet overlap, *pij* = proportion of item *i* relative to the total resources used by species *j*, *pik* = proportion of *i* relative to all resources used by species *k*, and *n* = the total number of resource items [59].

Trophic levels (TROPH) of co-existing native and alien fishes in the rivers sampled were determined from the diet composition data. These were analyzed following the quantitative routine of TROPHLab software [60]. In this case, the contribution of each food item based on the relative volume derived from the points method was used. The equation was applied as follows: $TLi=1\pm \sum^{\;}j\;\left(TLj\times DCij\right)$, where *TLj* = the fractional (i.e., non-integer) trophic level of the prey *j* [61] and *DCij* = the fraction of *j* in the diet of *i*.

### 2.9. Stable Isotopes Analysis

The preparation of samples for stable δ^15^N and δ^13^C analysis followed the method employed by Nakamura et al. [62] and Zulkifli et al. [63]. Muscle tissues from the dorsal parts of the fish were collected from selected representative fish sizes for each species in each of the three sites, such as the Gombak, Klang, and Langat Rivers. These were the sites from which sufficient numbers of native and alien fishes were obtained and initially subjected to stomach contents analysis. The muscle tissues were immediately stored at −20 °C until the final preparation. After that, the skin, bones, and scales were carefully separated from the muscle tissue and then washed with deionized water. The washed samples were then placed in Petri dishes before oven-drying at 60 °C for 24 to 48 h until constant weights were attained.

Excess lipids were removed from the dried and ground samples by treating them with a mixture of chloroform (analytical reagent grade; Fisher Chemical, Loughborough, UK) and methanol (AnaPur grade; Fisher Chemical, Hampton, NH, USA) (ratio 2:1) for 3 h. Following that, the samples were centrifuged at 2500 rpm for 10 min at a temperature of 4 °C with a high-speed refrigerated centrifuge (Sorvall, Ramsey, MN, USA). After discarding the supernatant, the resulting pellets were dried in a desiccator for at least 1 h. They were then fumed for 10 h with 12M HCl (analytical reagent (assay ≥ 37%); Sigma-Aldrich, St. Louis, MO, USA) to remove inorganic carbonates. Finally, excess acid was extracted using sodium hydroxide pellets for 3 h in a vacuum desiccator. The samples were dried for at least 1 h before sending for stable isotope analysis at the Department of Chemistry, Ministry of Science, Technology and Innovation, Petaling Jaya, Selangor, Malaysia. 

### 2.10. Statistical Analyses

Microsoft Excel (Office 365, Version 2016, Microsoft Corp., Berkshire, UK) was used for descriptive statistics to reveal the overall occurrence of individual fish species, fish species by origin (native or alien), and the percentage occurrences of fish families from sampled locations. Measured water quality parameters were compared across sites with a one-way analysis of variances (ANOVA) with IBM SPSS, ver. 22.0 (IBM Corp., Chicago, IL, USA). Before analysis, data were log-transformed to normalize them since they did not satisfy the conditions for a parametric test. Graphical presentation of the result was performed using Microsoft Excel (Office 365, ver. 2016, Microsoft Corp., Berkshire, UK). 

Principal Components Analysis (PCA) was used to extract the most crucial water quality parameters used to assess the relationships with fish occurrences using Canonical Correspondence Analysis (CCA). The IBM SPSS, ver. 22.0 (IBM Corp., Chicago, IL, USA) was used for this purpose. Further, the measured anthropogenic factors were related to fish occurrences using CCA. The analyses were done using the PAST (version 3.25) software.

Since the data were not normally distributed, non-parametric tests, such as Mann–Whitney U and Kruskal–Wallis H tests were used to compare stomach fullness indices, and δ^15^N and δ^13^C values of native and alien fishes. The closeness or overlap of the δ^15^N and δ^13^C values existing between the native and alien species was shown using the biplot of mean and standard deviations. Furthermore, to identify the trophic plasticity of the studied fishes, the δ^15^N and δ^13^C values of native and alien species, such as *Mystacoleucus obtusirostris* and *O. niloticus*, that occurred at least twice from the sampled rivers were compared. Except for TROPH, which was estimated using the TROPHLab software, all statistical analyses were done using Microsoft Excel 2016 (Office 365 ver. 2016) and IBM SPSS ver. 23 (IBM Corp., Chicago, IL, USA).

## 3. Results

### 3.1. Fish Checklist and Community Structures

A total of 20 fish species were recorded, out of which six are aliens (Table 1). Of all the sampled fishes, *O. niloticus*, *Poecilia reticulata*, and *Mystacoleucus obtusirostris* were the most occurring by percentage, occurring at 45.9%, 15.4%, and 12.7%, respectively. 

**Figure 2:**
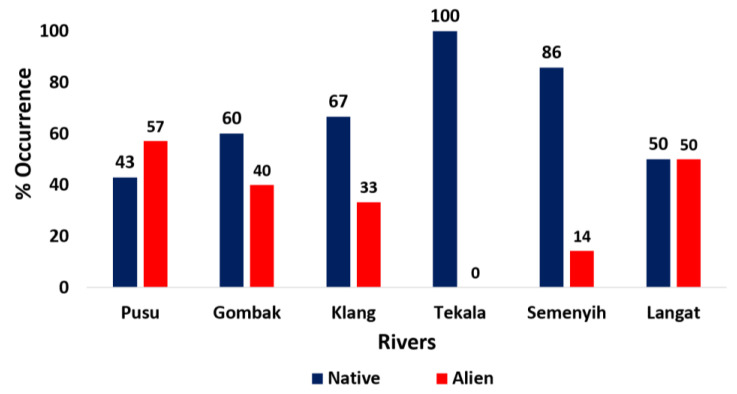
Overall occurrence (%) of individual fish species. Red and blue bars represent alien and native fish species, respectively.

The Langat River recorded the highest number of species (10 species), and the lowest numbers were recorded in the Gombak and Tekala Rivers (five species). With 45% and 35% occurrence in the six rivers, the order Cypriniformes and family Cyprinidae were generally the most occurring. Furthermore, based on occurrence in the sampled rivers by origin, the Pusu River had the highest percentage occurrence (57%) of alien fishes, followed by the Langat (50%) and Gombak Rivers (40%) (Figure 2). 

The Semenyih River recorded the highest fish richness (*Dmn* = 1.32), diversity ($H^{\prime }$= 1.80) and evenness (*J* = 0.87) indices, but with the lowest dominance index (*C* = 0.20). The Pusu River recorded the lowest diversity ($H^{\prime }$ = 0.94) and the highest dominance (*C* = 0.55) indices (Table 2). 

The Whittaker’s beta diversity index was highest (showing the greatest difference) for the Tekala vs. the Langat River (βw = 0.867), followed by the Pusu vs. the Klang River (βw = 0.750). It was lowest (showing the greatest similarity) for the Gombak vs. the Semenyih River (βw = 0.231), followed by the Pusu vs. the Gombak River (βw = 0.333) and the Tekala vs. the Semenyih River (βw = 0.385) (Table 3).

### 3.2. Water Quality Parameters and Anthropogenic Factors across Sites

Significant differences (*p* < 0.05) existed in the water quality parameters across sites except for depth. The highest means of temperature and pH were recorded at the Klang River (32.0 ± 0.21 °C) and Gombak River (7.48 ± 0.38), respectively. The Tekala River recorded the highest DO value (4.22 ± 0.54 mg/L), while TDS (90.82 ± 3.46 mg/L), salinity (0.07 ± 0.01 ppt), and conductivity (139.50 ± 1.29 μS/cm) were the highest in the Pusu River. The Semenyih River (40.82 ± 28.78 cm) was the deepest and recorded the highest turbidity (48.78 ± 17.77 NTU), while NO_3_^-^ was highest in the Gombak River (7.53 ± 2.76). However, NH_3_-N (0.99 ± 0.05 mg/L) and NO_2_^-^ (1.00 ± 0.51) recorded the highest measurement in the Langat River (Supplementary Material Table S3).

The scoring of anthropogenic factors around the sampling sites revealed that the Langat River, with a mean score of 3.80 ± 1.12, is likely the most exposed to the human elements. Next was the Pusu River with a mean score of 3.60 ± 1.74, while the Semenyih, Klang, Gombak, and Tekala Rivers scored 3.50 ± 1.58, 3.10 ± 1.69, 3.00 ± 1.40, and 2.50 ± 0.93, respectively.

### 3.3. Invasiveness Screening

AS-ISK v2 analyses revealed that *O. niloticus* had BRA and BRA+CCA scores of 24 each and certainty factors (CF) of 0.81 and 0.79, respectively. Moreover, *P. pardalis*, *P. disjunctivus*, *P. reticulata*, and *B. gonionotus* scored 36, 24, 19, and 5 for both BRA and BRA+CCA with CF values 0.90 and 0.89, 0.86 and 0.85, 0.83 and 0.82, and 0.88 and 0.86, respectively. Based on the ROC analysis, the minimum AUC value was close to one, indicating the predictive value of AS-ISK. Youden’s *J* statistic gave threshold values of 17.5 and 18.25 for BRA and BRA+CCA, respectively. Thus, except for *B. gonionotus*, which recorded a medium risk of invasion, values recorded for all other alien fishes indicate high invasion risks.

### 3.4. Relationships between Fish Occurrences vs. Water Quality Parameters and Anthropogenic Factors

Based on PCA and ordination plot analyses, three axes cumulatively explained 75.4% of the variation in the water quality parameters. Components with eigenvalues greater than one were considered as significant and thus extracted. Out of the 12 parameters measured, only six were retained based on the set criteria. Component one had strong loadings for temperature and DO, component two had strong loadings for PO_4_^3−^ and NO_3_^−^, while component two had strong loadings for NH_3_-N and NO_2_^−^ (Supplementary Material Table S4 and Figure S1).

**Figure 3:**
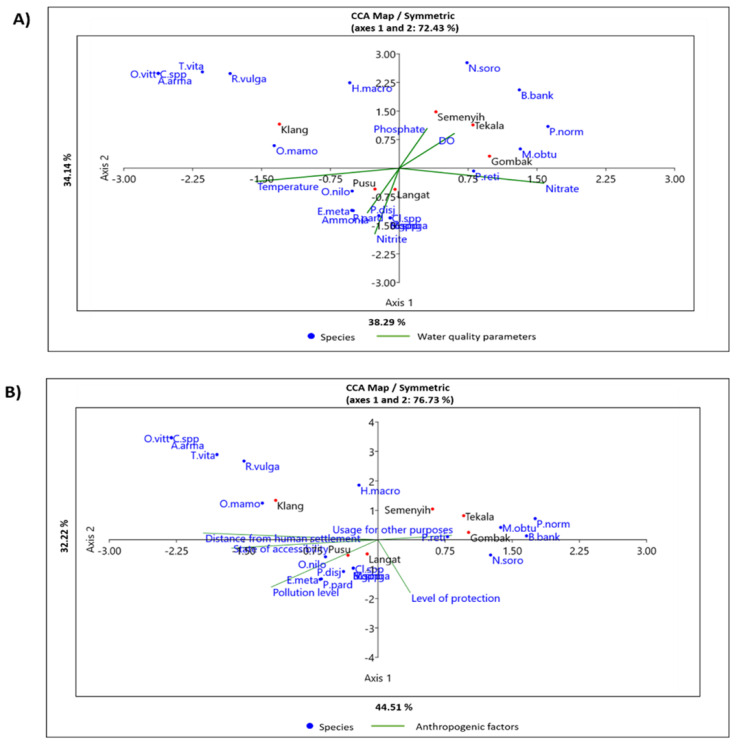
Ordination map from Canonical Correspondence (CC) analysis showing the relationships between fish occurrences; (**A**) water quality parameters; and (**B**) anthropogenic factors surrounding the six rivers sampled within the Klang Valley, Malaysia. Abbreviation used for fish species name as similarly reported in Table 1. Klang = Klang River; Semenyih = Semenyih River; Tekala = Tekala River; Gombak = Gombak River; Langat = Langat River; Pusu = Pusu River; DO = dissolved oxygen.

Ordination from CCA for water quality parameters vs. fish occurrence shows that the first three axes accounted for 87.68% (Supplementary Material Table S5). Further, the six variables with strong loadings from PCA showed that native species, such as *P. normani*, *M. obtusirostris*, *Neolissochilus soroides*, and *Barbodes banksi,* correlated positively with PO_4_^3−^ and DO and negatively with NH_3_-N and NO_2_^−^. Native species, such as *Esomus metallicus* and *Mytus singaringan*, with alien species, such as *O. niloticus*, *P. pardalis*, and *P. disjunctivus,* correlated positively with NH_3_-N, and NO_2_^−^, and negatively with PO_4_^3−^ and DO, while *P. reticulata,* which is also an alien species, associated positively with NO_3_^−^. Other native species, such as *Oxyeleotris marmorata*, *Osteochilus vitattus*, *Rasbora vulgaris*, *Trichopsis vitatta*, *Aplocheilus armatus*, and *Hampala macrolepidota*, and alien species, such as *B. gonionotus,* showed no clear relationship with any of the measured water quality parameters (Figure 3A). 

CCA for anthropogenic factors vs. fish occurrences showed that the first three axes accounted for 95.55% of the variances (Supplementary Material Table S6). High pollution levels and high ease of accessibility correlated positively with the occurrence of *O. niloticus*, *P. pardalis*, and *P. disjunctivus*, but negatively with native species, such as *M. obtusirostris* and *P. normani*, which also correlated with the usage for other purposes. The level of protection did not correlate with the occurrence of any of the species. In contrast, native species, such as *O. vittatus*, *T. vittata*, and *O. marmorata*, which were generally low in occurrences, did not show a clear pattern regarding the anthropogenic factors assessed (Figure 3B).

### 3.5. Feeding Intensity and Stomach Fullness Index

*Oreochromis niloticus* was the only species that occurred in abundant numbers for the three rivers assessed. Other alien species include *P. reticulata*, *P. disjunctivus*, and *P. pardalis*, and native species, such as *B. banksi*, *O. vittatus*, *O. marmorata*, *T. vittata*, and *H. macrolepidota,* were the only ones subjected to stable isotope analysis.

Since other species were limited in numbers, only *O. niloticus* (alien) was suitable for comparison (using stomach contents analysis) with native species, such as *M. obtusirostris*, *P. normani*, *R. vulgaris*, and *M. singaringan*. A descriptive summary of the fish sizes (*n* = 183) from Gombak, Klang, and Langat Rivers is presented in Table 4. Except for the Klang River, *O. niloticus* generally demonstrated the highest percentage of filled stomachs. The Kruskal–Wallis test revealed significant differences in the fullness indices (*H* = 17.057, df = 2, *p* = 0.000). The pairwise comparison revealed significant differences between *O. niloticus* and *P. normani* (*p* < 0.001) and *O. niloticus* and *M. obtusirostris* (*p* = 0.006). More so, the Mann–Whitney U test revealed significant differences between the fullness indices of *O. niloticus* and *R. vulgaris* (*U* = 109.500, *p* < 0.001) and *O. niloticus* and *M. singaringan* (*U* = 292.000, *p* < 0.001) for Klang and Langat Rivers, respectively (Table 5).

**Figure 4:**
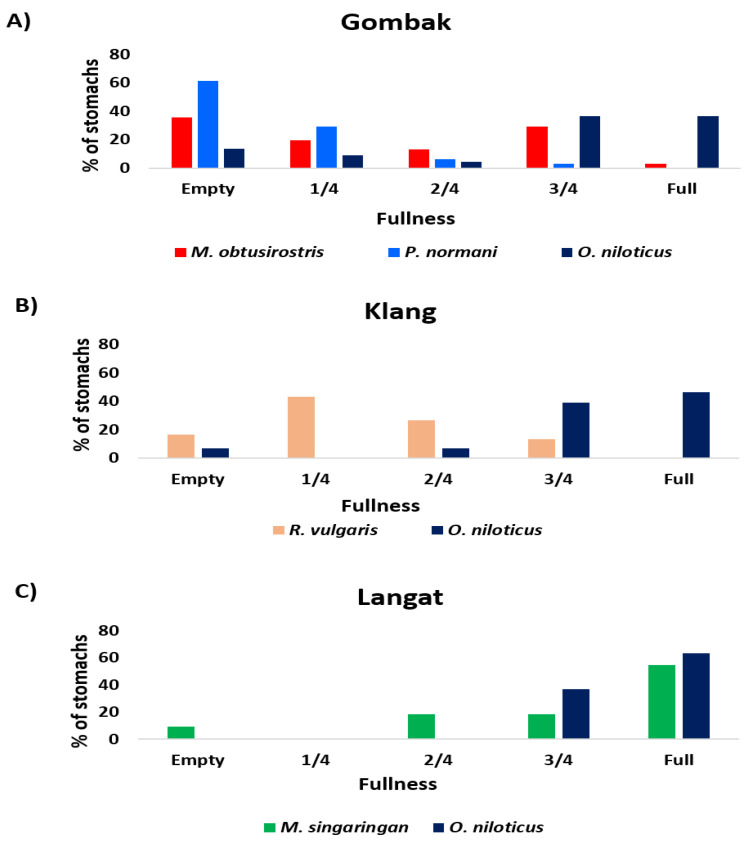
Stomach fulness level of fish species; (**A**) Gombak River; (**B**) Klang River; (**C**) Langat River. *Oreochromis niloticus* is the alien fish.

For the Gombak River, the level of stomach fullness for the three species considered indicated that *O. niloticus* had the highest percentage of full (36.36%) and 3/4 full stomachs (36.36%), while *P. normani* had the highest percentage of empty (61.29%) and 1/4 full stomachs (20.03%) (Figure 4). For the Klang River, the level of stomach fullness indicated that only *O. niloticus* recorded full stomachs (46.43%) with the highest 3/4 full stomachs (39.29%). In comparison, *R. vulgaris* had the highest percentage of empty (16.66%) and 1/4 full stomachs (43.33%). Meanwhile, for the Langat River, *O. niloticus* had the highest percentage of full (63.33%) and 3/4 full stomachs (36.67%), while *M. singaringan* had the highest percentage of empty (9.09%) and 3/4 full stomachs (18.18%).

### 3.6. Importance of the Food Items

For Gombak River, detritus recorded the highest percentage occurrence, volume, and preponderance. However, multicellular green algae recorded the highest percentage occurrence (90.00%), volume (21.56%), and preponderance (23.48%) in the stomachs of *M. obtusirostris*. Multicellular green algae were also the highest by occurrence (100%), volume (11.71%), and preponderance (14.56%) for *P. normani*. For *O. niloticus*, diatoms were the most occurring (100%), highest by volume (27.04%), and preponderance (27.04%) (Table 6).

For the Klang River, insect parts were the highest by occurrence (100%), volume (44.13%), and preponderance (49.79%) in the stomachs of *R. vulgaris*. For *O. niloticus*, cyanobacteria, green algae (unicellular and multicellular), diatoms, detritus, and mud occurred in 100% of the stomachs, while diatoms (17.47%) followed detritus (40.00%) as the most important by volume. The same pattern was observed by preponderance for diatoms (18.98%) and detritus (43.89%), respectively (Table 7).

Detritus was generally the highest by occurrence, volume, and preponderance in the two fish species considered for the Langat River. Multicellular green algae and plant parts also occurred in all the non-empty stomachs (100%) of *M. singaringan*, while worms were next in importance by volume (15.67%) and preponderance (14.25%). Mud occurred in all the non-empty stomachs (100%) of *O. niloticus*, while detritus was the most important by volume (24.76%) and by preponderance (29.01%), followed by worms by volume (18.17%) and preponderance (18.36%) (Table 8). Some examples of the stomach contents encountered in fish samples are presented in the Supplementary Material Figure S2.

### 3.7. Diet Overlap and Trophic Level

Except for *R. vulgaris* vs. *O. niloticus* in the Klang River, the Morisita–Horn index (C_H_) for alien vs. native species indicated a significant diet overlap (C_H_ > 0.6) for all the pairs of species in each river (Supplementary Material Table S7). In the Gombak River, *M. obtusirostris* (2.12 ± 0.15) had the highest TROPH value, while *R. vulgaris* (2.6 ± 0.28) and *M. singaringan* (2.33 ± 0.20) had higher values for the Klang and Langat Rivers, respectively (Supplementary Material Table S8).

### 3.8. Stable Isotope Analysis

**Figure 5:**
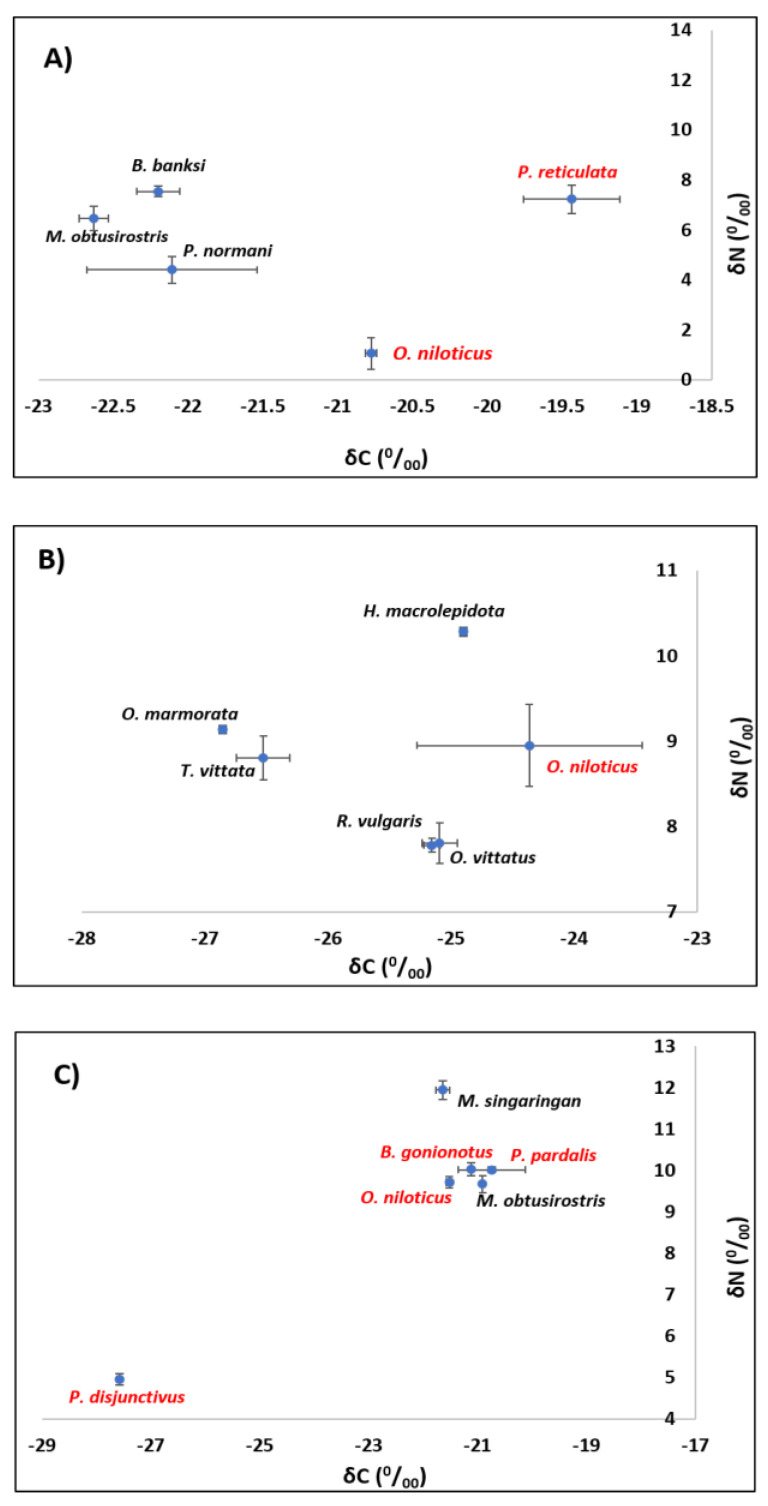
Scatter plot of mean δ^13^C and δ^15^N (± SD) for the native (black) and alien (red) fish species from the (**A**) Gombak, (**B**) Klang, and (**C**) Langat Rivers.

The positioning and overlap in the δ^15^N and δ^13^C for native and alien fish species are presented in Figure 5A–C for the Gombak, Klang and Langat Rivers, respectively. These are scatter plots that indicate the positioning of native and alien fish species recorded based on their stable δ^15^N and δ^13^C signatures. From the scatterplots, the greatest overlap between alien and native fish species was recorded for Klang River, where *O. niloticus* overlapped with both *R. vulgaris* and *O. vittatus* based on δ^13^C values. There was also an overlap between *O. niloticus* and both *O. marmorata* and *T. vittata* based on their δ^15^N values. The Kruskal–Wallis test revealed the existence of significant differences in the δ^15^N (*H* = 13.524, df = 4, *p* = 0.009) and δ^13^C (*H* = 12.900, df = 4, *p* = 0.012) of fish species from the Gombak River. For the δ^15^N values, pairwise comparisons showed that except for the significant differences observed between *O. niloticus* and *B. banksi* (*p* = 0.010), there were no significant differences (*p* > 0.05) between all other pairs of fish species analyzed. For the δ^13^C values, pairwise comparisons showed that except for the significant differences observed between *M. obtusirostris* and *P. reticulata* (*p* = 0.010), there were no significant differences (*p* > 0.05) between all other pairs of fish species analyzed. 

For the Klang River, the Kruskal–Wallis test revealed the existence of significant differences in the δ^15^N (*H* = 17.115, df = 5, *p* = 0.004) and δ^13^C (*H* = 14.327, df = 5, *p* = 0.014) for fish species from the Klang River. For the δ^15^N values, pairwise comparisons showed that except for the significant differences observed between *H. macrolepidota* and *R. vulgaris* (*p* = 0.015), and *H. macrolepidota* and *O. vittatus* (*p* = 0.030), there were no significant differences (*p* > 0.05) between all other pairs of fish species analyzed. For the δ^13^C values, pairwise comparisons showed that except for the significant differences observed between *O. marmorata* and *O. niloticus* (*p* = 0.031), there were no significant differences (*p* > 0.05) between all other pairs of fish species analyzed.

For the Langat River, the Kruskal–Wallis test revealed the existence of significant differences in the δ^15^N (*H* = 15.339, df = 5, *p* = 0.009) and δ^13^C (*H* = 12.202, df = 5, *p* = 0.032) for fish species from the Langat River. For the δ^15^N values, pairwise comparisons showed that except for the significant differences observed between *P. disjunctivus* and *M. singaringan* (*p* = 0.01), there were no significant differences (*p* > 0.05) between all other pairs of fish species analyzed. For the δ^13^C values, pairwise comparisons showed that except for the significant differences observed between *M. obtusirostris* and *P. disjunctivus* (*p* = 0.019), there were no significant differences (*p* > 0.05) between all other pairs of fish species analyzed.

For *M. obtusirostris* sampled across the Gombak and Langat Rivers, the Mann–Whitney U test revealed no significant differences (*p* > 0.05) between their δ^15^N and δ^13^C values. According to the Kruskal–Wallis H test, *O. niloticus* sampled across the Klang, Gombak and Langat Rivers showed significant differences between their δ^15^N (*H* = 9.346, df = 2, *p* < 0.009) and δ^13^C (*H* = 9.379, df = 2, *p* < 0.009) values. For the δ^15^N values, pairwise comparisons showed a significant difference in the δ^15^N values between Gombak and Langat Rivers (*p* = 0.007). Moreover, a significant difference in the δ^13^C values was observed between the Gombak and Klang Rivers (*p* = 0.010).

## 4. Discussion

The present study set out with the aim of assessing the invasion risks of the identified alien fishes using AS-ISK and the fish community structures of selected rivers within the Klang Valley, Malaysia. More so, the fish occurrences were related to water quality parameters and anthropogenic factors. The stomach contents of co-existing native and alien fishes from three of the six rivers were also assessed, while stable isotope analyses, through the assessment of δ^15^N and δ^13^C values in fish muscle tissues, were carried out.

The results of this study revealed that, except for the Tekala River, alien fishes were recorded for all of the sites, with the most frequently occurring alien species being *O. niloticus*. Moreover, the sight of breeding nests belonging to this species in the Klang River confirms that this species had established a breeding population in this river. In line with the findings of this study, Shuai et al. [64] reported an increase in the relative abundance of *O. niloticus*, which is a non-native species, in a large subtropical river in China. Therefore, the ability of this alien species to successfully breed and multiply over time is confirmed, and this further explains the result of this study. 

Similar to some other high-risk alien ornamental fish species recorded from pet stores in the Klang Valley, Malaysia, which had been earlier screened using the FISK v2 software [4], *O. niloticus* was labeled as invasive. Similarly, the high invasion risk of *O. niloticus* had been established in other regions too, including the Iberian Peninsula [65], South Africa [66], and China [64], where they have negatively impacted the native fish species and the environment. Consequently, the negative impacts that stem from the establishment of invasive species, such as *O. niloticus,* are also predicted for the water bodies that were the subject of this study.

The comparatively higher fish species richness, evenness, and lower dominance exhibited by the Semenyih and Tekala Rivers coincided with the low or no occurrence of alien fishes in these sites. More so, it indicates that the absence of alien fishes may have provided a better environment for native fishes to exist and flourish in these rivers. Generally, low fish diversity was commensurate with low richness and high dominance. Shannon’s diversity index recorded in this study for the Pusu River is lower than that reported by Jalal et al. [31]. The number of alien fish species recorded in their study (*n* = 2) was lower than that recorded in the present study (*n* = 4), indicating that additional alien fishes may have been introduced from time to time. These species have succeeded in establishing breeding populations in the Pusu River. Specifically, *O. niloticus* was the dominant fish species in the Pusu River instead of *B. schwanenfeldii*, which is a native fish species that was earlier reported to be the most prevalent in the river.

This study discovered that alien fishes have shadowed the native ones in rivers with high NH_3_-N, TDS, conductivity and salinity, and low DO levels and water depths. More so, most of the native fishes assessed have shown the ability to thrive better in water bodies with higher DO levels, low levels of NH_3_-N, and the absence or low diversity of alien species as was observed in the Tekala and Semenyih Rivers. Moreover, the alien fish species displayed a comparatively higher abundance in rivers that recorded high TDS, conductivity, salinity, and NH_3_-N as observed in the Pusu and Klang Rivers. Fishes generally prefer water conditions of adequate physical and chemical characteristics [67]. However, many invasive fish species possess some special characteristics that make them highly sought after [68]. For example, the ability to survive and grow at low levels of dissolved oxygen and high levels of ammonia. More so, the characteristics of these alien fish species may contrast with those of their native counterparts, which may not be rugged enough to withstand a similar condition. Thus, according to the present study, the alien fishes tend to be more rugged and could flourish in these water bodies to the detriment of their native counterparts.

The anthropogenic factors indicate a possible contribution of human influence to the community and environmental indices such that the potential of alien fishes to invade the waterbodies is amplified. For example, the Langat River seems to be more exposed to anthropogenic influences. Furthermore, activities, such as housing construction, waste dumping, and land clearing, have been reported around the Langat and Pusu Rivers [69]. Public knowledge of the possible negative environmental impacts of human activities, including the introduction of alien fishes, is essential for effective conservation and management of inland freshwater [70]. In the present study, these rivers were characterized by high NH_3_-N and low DO concentrations, indicating that native species could be outcompeted since their alien counterparts are better able to withstand the poor water quality conditions. 

*Oreochromis niloticus*, an omnivorous species, ingests zooplankton, phytoplankton, and debris present in rivers, resulting in competition for food and space with the native fishes [71]. Moreover, *O. niloticus* reportedly replaced native species in Thailand after excessive reproduction success [72]. They also predate on juveniles and eggs of native species and disrupt the habitat by grazing on benthic algae and detritus [73]. Paradoxically, due to its excellent culture characteristics, *O. niloticus* has been widely introduced to several countries across the globe for aquaculture improvement and to augment capture fisheries [74]. This fish was introduced into Malaysia for aquaculture and may have escaped from culture facilities into native waters due to natural disasters, such as flooding [3]. 

Similar to its other counterparts identified in this study, *O. niloticus* is invasive, and this study represents the first attempt to risk-assess this species for Peninsular Malaysia. Previous risk assessment studies, including Ellender et al. [75] for South Africa, and Perdikaris et al. [76] for Greece, have also found *O. niloticus* invasive. *Pterygoplichthys disjunctivus* was also found to pose a high invasion risk in South Africa [68], while *P. pardalis*, *P. disjunctivus*, and *P. reticulata* were invasive in Mexico [77]. 

With the highest percentage of full stomachs and significantly higher stomach fullness indices throughout the sampling sites, *O. niloticus* displayed the best feeding ability. Previous reports have shown that some native species exploit similar food with the *O. niloticus*, which can outcompete them, making them shift their preferences to less preferred resources [78]. This can consequently impact negatively on their growth and condition in the ecosystem [16,75,79].

Furthermore, diet overlap between pairs of alien and native fish species significantly showed that they essentially exploit similar food resources. This signifies competition between these fish species, with *O. niloticus* being more rugged and plastic [80]. Broad niche overlap of *O. niloticus* with native species in South Africa was also reported by Zengeya et al. [81,82]. However, in an experimental study, Ahmad et al. [83] recorded lower levels of overlap between *O. niloticus* and native small indigenous fish species of south Asia, such as mola (*Amblypharyngodon mola*), chela (*Chela cachius*), and punti (*Puntius sophore*). The omnivorous nature of the species considered in that study and the availability of alternative food resources may be responsible for the difference.

The TROPH values of both alien and native fishes indicated that they are all positioned within the same trophic level in the three rivers. From the TROPH values recorded in this study, *O. niloticus* from the Gombak and Klang Rivers are herbivorous. However, the values indicate that they are omnivorous with a preference for food of plant origin in the Langat River. In line with the outcome of the current study, *O. niloticus* had also been regarded as omnivorous and planktivorous, feeding mainly on green algae, diatoms, cyanobacteria, and detritus [84,85].

The results of this study indicate similarities in the trophic preferences of both native and alien fishes and the trophic plasticity of *O. niloticus* as opposed to its native counterpart *M. obtusirostris* [86]. Therefore, an alien species, such as *O. niloticus,* could explore food at a wider trophic range giving it the ability to outcompete the native species of narrower δ^15^N and δ^13^C values [87,88]. Diet overlaps between *O. niloticus* and the native fishes were found based on stomach contents analysis. The result from stable isotope analyses gave an insight into the statistical similarities and differences in the stable isotope signatures of the fish species; however, it may not be sufficient to conclude that the alien and native species have overlapping isotopic niches.

## 5. Conclusions

This study indicates that compared to native fish species, alien fish species likely benefited from the impacts of anthropogenic activities in their surrounding habitats, while their plasticity in feeding habits might help them to further invade, survive, and dominate. The potential ecological impacts on native fishes in the rivers of Klang Valley, Malaysia was, therefore, revealed.

## Figures and Tables

**Table 1:** Checklist and number of individuals of fish species recorded from sampled rivers within the Klang Valley, Malaysia.

Order	Family	Species	Abbr	Number of Individuals Per Site							% of Occurrences
				P	G	K	T	S	L	Total	
Cyprinidontiformes	Poecilidae	*Poecilia reticulata* Peters, 1859 *	P.reti	55	75	2	0	0	8	140	15.4
Cyprinidontiformes	Aplocheilidae	*Aplocheilus armatus* (van Hasselt 1823)	A.arma	0	0	2	0	0	0	2	0.2
Cypriniformes	Cyprinidae	*Mystacoleucus obtusirostris* (Valenciennes, 1842)	M.obtu	6	64	0	18	4	23	115	12.7
Cypriniformes	Cyprinidae	*Poropuntius normani* Smith, 1931	P.norm	3	49	0	4	12	0	68	7.5
Cypriniformes	Cyprinidae	*Hampala macrolepidota* Kuhl and Van Hasselt, 1823	H.macro	0	0	1	1	0	0	2	0.2
Cypriniformes	Cyprinidae	*Osteochilus vittatus* (Valenciennes, 1842)	O.vitt	0	0	4	0	0	0	4	0.4
Cypriniformes	Cyprinidae	*Barbodes banksi* (Herre, 1940)	B.bank	0	2	0	4	4	0	10	1.1
Cypriniformes	Cyprinidae	*Neolissochilus soroides* (Duncker, 1904)	N.soro	0	0	0	0	1	0	1	0.1
Cypriniformes	Cyprinidae	*Barbonymus gonionotus* (Bleeker, 1849) *	B.goni	0	0	0	0	0	2	2	0.2
Cypriniformes	Danionidae	*Esomus metallicus* Ahl, 1923	E.meta	4	0	0	0	0	0	4	0.4
Cypriniformes	Danionidae	*Rasbora vulgaris* Duncker, 1904	R.vulga	0	0	48	5	8	0	61	6.7
Perciformes	Cichlidae	*Oreochromis niloticus* (Linnaeus, 1758) *	O.nilo	272	27	46	0	2	69	416	45.9
Perciformes	Cichlidae	*Oreochromis* sp. *	C.spp	0	0	2	0	0	0	2	0.2
Perciformes	Oshpronemidae	*Trichopsis vittata* (Cuvier, 1831)	T.vita	0	0	6	0	1	0	7	0.8
Perciformes	Eleotridae	*Oxyeleotris marmorata* (Bleeker, 1852)	O.mamo	0	0	1	0	0	1	2	0.2
Siluriformes	Loricariidae	*Pterygoplichthys pardalis* (Castelnau, 1855) *	P.pard	28	0	0	0	0	1	29	3.2
Siluriformes	Loricariidae	*Pterygoplichthys disjunctivus* (Weber, 1991) *	P.disj	8	0	0	0	0	19	27	3.0
Siluriformes	Bagridae	*Mystus singaringan* (Bleeker, 1846)	M.singa	0	0	0	0	0	11	11	1.2
Siluriformes	Bagridae	*Hemibagrus* sp.	H.spp	0	0	0	0	0	1	1	0.1
Siluriformes	Clariidae	*Clarias* sp.	Cl.spp	0	0	0	0	0	3	3	0.3
**Total Fish Individuals**				**376**	**217**	**112**	**32**	**32**	**138**	**907**	**100**

P = Pusu River, G = Gombak River, K = Klang River, T = Tekala River, S = Semenyih River, L = Langat River, (*) = alien species, Abbr. = abbreviation.

**Table 2:** Fish species richness, diversity, evenness, and dominance indices of selected rivers in the Klang Valley, Malaysia.

River	*Dmn*	*H’*	*J*	*C*
Pusu	0.361	0.944	0.485	0.551
Gombak	0.339	1.366	0.849	0.273
Klang	0.850	1.304	0.594	0.358
Tekala	0.884	1.242	0.772	0.373
Semenyih	1.315	1.801	0.866	0.198
Langat	0.851	1.537	0.667	0.307

*Dmn =* Menhinnick’s richness index, *H*′ = Shannon’s diversity index, *J* = Pielou’s evenness index and *C* = Simpson’s dominance index.

**Table 3:** Pairwise comparison from Whittaker’s beta diversity indices. Lower values show higher similarity.

River	Pusu	Gombak	Klang	Tekala	Semenyih	Langat
Pusu		0.333	0.750	0.667	0.467	0.412
Gombak			0.714	0.400	0.231	0.600
Klang				0.714	0.529	0.684
Tekala					0.385	0.867
Semenyih						0.667
Langat						

**Table 4:** Summary of total length (cm) and percentage of full stomachs for native and alien fish samples used for stomach contents analysis.

River	Species	Mean ± SD	*n*	Min	Max	%FS
Gombak	*Mystcoleucus obtusirotris*	9.69 ± 1.46	31	7.30	13.00	64.50
	*Poropuntius normani*	7.88 ± 2.20	31	6.60	13.40	41.90
	*Oreochromis niloticus* *	6.78 ± 0.91	22	5.30	8.90	86.40
Klang	*Rasbora vulgaris*	11.42 ± 4.85	30	5.00	21.50	92.90
	*Oreochromis niloticus* *	8.74 ± 0.58	28	7.70	9.80	83.30
Langat	*Mystus singaringan*	14.15 ± 1.38	11	10.90	16.50	90.90
	*Oreochromis niloticus* *	10.51 ±1.92	30	6.50	14.30	100.00

%FS = percentage of filled stomachs. * indicates alien species.

**Table 5:** Mean comparison of fullness index among alien and native species.

River	Species	Mean ± SD
Gombak	*Mystacoleucus obtusirostris*	0.38 ± 0.26 ^a^
	*Poropuntius normani*	0.23 ± 0.15 ^a^
	*Oreochromis niloticus* *	1.05 ± 0.72 ^b^
Klang	*Rasbora vulgaris*	0.29 ± 0.25 ^a^
	*Oreochromis niloticus* *	1.26 ± 0.95 ^b^
Langat	*Mystus singaringan*	0.35 ± 0.30 ^a^
	*Oreochromis niloticus* *	1.17 ± 0.55 ^b^

* indicates alien species. Means with different superscripts indicate significant differences.

**Table 6:** Food categories from the stomach contents of fish species sampled from the Gombak River.

	*Mystacoleucus obtusirostris* *n* = 31			*Poropuntius normani* *n* = 31			*Oreochromis niloticus* * n = 22		
Food Category	FO (%)	V (%)	IP (%)	FO (%)	V (%)	IP (%)	FO (%)	V (%)	IP (%)
Cyanobacteria	70.00	2.68	2.27	53.85	12.22	8.18	94.74	6.46	6.56
Multicellular green algae	90.00	21.56	23.48	100.00	11.71	14.56	52.63	3.65	1.06
*Cladophora*	0.00	0.00	0.00	0.00	0.00	0.00	21.05	1.57	0.35
Other multicellular algae	90.00	21.56	23.48	100.00	11.71	14.56	31.58	2.08	0.70
Unicellular green algae	0.00	0.00	0.00	23.08	0.61	0.09	84.62	7.42	4.80
*Chlamydomonas*	0.00	0.00	0.00	0.00	0.00	0.00	42.11	1.52	0.68
*Chroococcus*	0.00	0.00	0.00	7.69	0.31	0.03	78.95	3.60	3.04
*Synedra*	0.00	0.00	0.00	0.00	0.00	0.00	57.89	1.52	0.94
Other unicellular algae	0.00	0.00	0.00	15.38	0.31	0.06	15.79	0.79	0.13
*Euglena*	0.00	0.00	0.00	0.00	0.00	0.00	36.84	0.73	0.29
Red algae	10.00	2.82	0.17	23.08	1.12	0.32	0.00	0.00	0.00
*Compsopogon*	5.00	2.26	0.14	0.00	0.00	0.00	0.00	0.00	0.00
Other red algae	5.00	0.56	0.03	23.08	1.12	0.32	0.00	0.00	0.00
Detritus	100.00	48.27	58.39	100.00	52.85	65.71	100.00	45.42	48.63
Insect part	50.00	13.53	8.18	30.77	8.35	3.19	0.00	0.00	0.00
Diatoms	70.00	4.09	3.46	84.62	4.07	4.29	100.00	27.04	28.95
Worms	0.00	0.00	0.00	0.00	0.00	0.00	10.53	0.11	0.01
Unidentified	0.00	0.00	0.00	0.00	0.00	0.00	5.26	0.11	0.01
Plant parts	10.00	0.35	0.04	15.38	5.70	1.09	0.00	0.00	0.00
Mud	50.00	6.62	4.01	61.54	3.36	2.57	100.00	9.05	9.69
Zooplankton	5.00	0.07	0.00	0.00	0.00	0.00	0.00	0.00	0.00
Rotifer	5.00	0.07	0.00	0.00	0.00	0.00	0.00	0.00	0.00

* indicates alien species, FO (%) = percentage frequency of occurrence, V (%) = percentage volume, and IP (%) = percentage index of preponderance.

**Table 7:** Food categories from the stomach contents of fish species sampled from Klang River.

	*Rasbora vulgaris* *n* = 30			*Oreochromis niloticus* * n = 28		
Food Category	FO (%)	V (%)	IP (%)	FO (%)	V (%)	IP (%)
Cyanobacteria	16.00	0.45	0.08	100.00	3.57	3.88
Multicellular green algae	76.00	4.11	3.42	100.00	11.89	11.13
*Cladophora*	4.00	0.15	0.01	14.81	1.92	0.31
Other multicellular algae	76.00	3.96	3.41	100.00	9.97	10.83
Unicellular green algae	0.00	0.00	0.00	100.00	5.99	5.83
*Chlamydomonas*	0.00	0.00	0.00	11.11	0.10	0.01
*Chroococcus*	0.00	0.00	0.00	100.00	4.61	5.01
*Closterium*	0.00	0.00	0.00	59.26	1.25	0.80
Other unicellular green algae	0.00	0.00	0.00	3.70	0.03	0.00
Diatoms	8.00	0.22	0.02	100.00	17.47	18.98
Red algae	4.00	0.60	0.03	3.70	0.03	0.00
*Compsopogon*	0.00	0.00	0.00	0.00	0.00	0.00
Other red algae	4.00	0.60	0.03	3.70	0.03	0.00
Detritus	100.00	38.82	43.96	100.00	40.40	43.89
Unidentified	8.00	3.29	0.30	14.81	1.04	0.17
Plant parts	28.00	6.66	2.11	70.37	6.50	4.97
Mud	0.00	0.00	0.00	100.00	8.99	9.76
Zooplankton	0.00	0.00	0.00	33.3	0.67	0.10
Unidentified zooplankton	0.00	0.00	0.00	14.81	0.20	0.03
Rotifer	0.00	0.00	0.00	11.11	0.30	0.04
*Daphnia*	0.00	0.00	0.00	14.81	0.17	0.03
Fish egg	8.00	0.82	0.07	11.11	0.24	0.03
Fish scale	4.00	0.60	0.03	11.11	0.07	0.01
Worms	4.00	0.30	0.01	66.67	1.21	0.88
Insect part	100.00	44.13	49.97	18.52	1.92	0.39

* indicates alien species, FO (%) = percentage frequency of occurrence, V (%) = percentage volume, and IP (%) = percentage index of preponderance.

**Table 8:** Food categories from the stomach contents of fish species sampled from Langat River.

	*Mystus singaringan* *n* = 11			*Oreochromis niloticus* * n = 30		
Food Category	FO (%)	V (%)	IP (%)	FO (%)	V (%)	IP (%)
Cyanobacteria	60.00	1.22	0.95	60.00	1.31	0.95
Multicellular green algae	100.00	8.24	10.70	96.67	18.12	17.38
*Cladophora*	0.00	0.00	0.00	73.33	11.84	10.52
Other algae	100.00	8.24	10.70	90.00	6.28	6.85
Unicellular green algae	0.00	0.00	0.00	80.00	2.64	2.18
*Colestrium*	0.00	0.00	0.00	6.67	0.06	0.00
*Chroococcus*	0.00	0.00	0.00	3.33	0.03	0.00
Other unicellular green algae	0.00	0.00	0.00	70.00	2.56	2.17
Red algae	70.00	11.70	5.95	60.00	7.78	3.40
*Compsopogon*	10.00	4.88	0.63	36.67	6.28	2.79
Other red algae	60.00	6.82	5.31	33.33	1.50	0.61
Detritus	100.00	27.47	35.68	96.67	24.76	29.01
Insect part	70.00	13.94	12.67	30.00	1.89	0.69
Diatoms	20.00	0.41	0.11	53.33	0.94	0.61
Worms	70.00	15.67	14.25	83.33	18.17	18.36
Unidentified	10.00	1.83	0.24	16.67	1.06	0.21
Plant parts	100.00	11.39	14.80	96.67	13.50	15.83
Mud	70.00	3.36	3.05	100.00	9.36	11.35
Zooplankton	40.00	2.85	0.95	6.67	0.44	0.04
Unidentified zooplankton	30.00	1.63	0.63	6.67	0.44	0.04
Rotifer	20.00	1.22	0.32	0.00	0.00	0.00
Fish scales	30.00	1.53	0.59	3.33	0.03	0.00
Leech	10.00	0.41	0.05	0.00	0.00	0.00

* indicates alien species, FO (%) = percentage frequency of occurrence, V (%) = percentage volume, and IP (%) = percentage index of preponderance.

## Data Availability

All data related to this study will be provided on a request sent to the corresponding or first author.

## Supplementary Materials

The following are available online at https://www.mdpi.com/article/10.3390/ani11113152/s1, Figure S1: Ordination plot from Principal Components Analysis for water quality parameters from the sampling locations. Rotation Method: Varimax with Kaiser Normalization, Figure S2: Stomach contents of the collected fishes. This file contains some examples of the (A) Insect; (B) Zooplankton; (C) Worm; (D) Fish scale; (E) Detritus; (F) Mud particles; (G) Unicellular algae; (H) Diatoms; (I) Cyanobacteria; (J) Green algae; (K) Red algae; and (L) Plant parts that encountered in the stomachs of fishes. Magnifications range from 4× to 40×, Table S1: Environmental conditions and characteristics of the sampling points. This file contains detailed information on the environmental conditions, characteristics and coordinates of the rivers, Table S2: Criteria for the measurement of anthropogenic characteristics at each sampling site within Klang Valley, Malaysia, Table S3: Minimum, maximum and mean ± SD values of measured water quality parameters from selected rivers within Klang Valley, Malaysia. Rows with different superscripts indicate significant difference (*p* < 0.05) between the means of the log-transformed data, Table S4: Loadings from Principal Components Analysis for water quality parameters from the sampled rivers. Extraction was based on eigenvalues > 1. Numbers in bold indicate parameters with high loadings, Table S5: Eigen values and percentage variance of Canonical Correspondence Analysis for water physicochemical parameters from the sampled rivers., Table S6: Eigenvalues and percentage variance of Canonical Correspondence Analysis for anthropogenic factors surrounding the sampled rivers, Table S7: Diet overlaps between fish species from Gombak, Klang, and Langat Rivers based on Morisita-Horn index. CH = Morisita-Horn’s diet overlap index. * indicate alien species, Table S8: TROPH of native and alien fish species from Gombak, Klang, and Langat Rivers. *indicate alien species.
