# Supplementary material for: Invasion Risk and Potential Impact of Alien Freshwater Fishes on Native Counterparts in Klang Valley, Malaysia
Source: Animals (Basel). 2021 Nov 4;11(11):3152. doi: 10.3390/ani11113152 (PMC8614500; doi:10.3390/ani11113152)
Supplement: Supplementary file 1 [file animals-11-03152-s001.zip › animals-1390400-supplementary.pdf]

Supplementary Material Table S1

Environmental conditions and characteristics of the sampling points. This file contains detailed information on the environmental conditions, characteristics and coordinates of the rivers.

River	Environmental conditions and characteristics	Coordinates
Pusu	A tributary of the Gombak River. This river is located downstream close to the International Islamic University Malaysia campus and flows moderately over a sandy substrate with murky waters and dense canopy cover. The riverbank is also covered with tall grasses.	3 °15' 0" N, 101 °43' 44.4" E
Langat	This river is located upstream, close to construction sites and residential areas, and has a mixture of muddy and sandy bottoms with moderate water flow, little or no canopy, and the riverbank is covered with grasses.	3 °7' 4.8" N, 101 °49' 15.6" E
Semenyih	This river is fast flowing over a sandy bottom with a bridge that runs over the river. The riverbank is also covered with vegetations.	3 °0' 7.2" N, 101 °52' 30" E
Gombak	This river is situated upstream around the Kampung Sungai Chinchin and has a sandy bottom with some rocks scattered around. The water is clear with a moderate flow and the vegetations at the banks also provide canopies at some sections of the river.	3 °15' 18" N,101 °43' 22.8" E
Klang	The sampling location is upstream of the river around the Klang Gates Dam. The water is shallow and clear, running over a sandy bottom in which tilapia breeding nests were visibly buried.	3 °13' 55.2" N, 101 °45' 3.6" E
Tekala	This river is a tributary of the Semenyih River and is located adjacent to the Tekala River Recreation Forest. The water is clear running moderately over a combination of sandy and rocky bottoms and enjoys a massive canopy from the trees and shrubs around. The riverbank is also covered with grasses.	3 °3' 26.9" N, 101 °52' 23.3" E

Supplementary Material Table S2

Criteria for the measurement of anthropogenic characteristics at each sampling site within Klang Valley, Malaysia.

Score	Distance from human settlement	State of accessibility	Level of protection	Pollution level	Usage for other purposes
1	> 200 m	Hidden, no visible access road, not easily accessible.	None at all	No visible pollutants or waste materials.	None visible
2	150 – 199 m	Visible access road, non-motorable, not easily accessible.	Presence of restrictive structures like gates	Non-dumpsite, visible but degradable wastes	Research
3	100 – 149 m	Non-motorable road, easily accessible.	No visible warnings but with community alertness	Dumpsite visible but degradable materials present.	Recreation /fishing
4	50 – 99 m	Motorable road, easily accessible.	Only visible warnings but no community interference	Dumpsite visible, but non-degradable waste materials present.	Religious activities
5	< 49 m	In the open, by road side, directly and easily accessible.	Visible warnings and signs with community alertness	Dumpsite, visible industrial effluents and visible non-degradable solid wastes.	Domestic water source

Supplementary Material Table S3

Minimum, maximum and mean ± SD values of measured water quality parameters from selected rivers within Klang Valley, Malaysia.

Rows with different superscripts indicate significant difference (p < 0.05) between the means of the log-transformed data.

Parameter		River					
		Pusu	Gombak	Klang	Tekala	Semenyih	Langat
Temperature (℃)	Min	28.00	26.60	31.60	25.50	27.60	30.70
	Max	30.10	31.70	32.20	26.00	33.20	30.90
	Mean ± SD	28.9 ± 0.84[a]	29.9 ± 2.44[ab]	32.0 ± 0.21[c]	25.7 ± 0.16[d]	29.89 ± 2.20[ac]	30.80 ± 0.11[bc]
pH (1-14)	Min	7.11	6.86	5.94	5.66	6.52	7.02
	Max	7.38	7.98	6.34	6.42	8.00	7.64
	Mean ± SD	7.28 ± 0.09[ab]	7.48 ± 0.38[b]	6.13 ± 0.12[c]	5.85 ± 0.22[c]	6.97 ± 0.44[a]	7.31 ± 0.22[ab]
Dissolved oxygen (mg/L)	Min	1.79	3.00	2.14	3.31	1.83	2.57
	Max	2.46	1.33	3.85	5.03	3.12	3.34
	Mean ± SD	2.10 ± 0.26[a]	2.42 ± 0.53[ab]	2.83 ± 0.61[b]	4.22 ± 0.54[c]	2.41 ± 0.49[ab]	2.90 ± 0.31[b]
Total dissolved solids (mg/L)	Min	86.45	36.40	14.95	10.40	19.50	59.15
	Max	95.12	46.15	29.25	11.05	24.70	72.15
	Mean ± SD	90.82 ± 3.46[a]	42.54 ± 3.09[b]	26.74 ± 4.67[c]	10.83 ± 0.33[d]	21.74 ± 1.87[e]	62.96 ± 3.95[f]
Salinity (ppt)	Min	0.06	0.02	0.01	0.01	0.01	0.04
	Max	0.07	0.03	0.02	0.01	0.02	0.05
	Mean ± SD	0.07 ± 0.01[a]	0.03 ± 0.00[b]	0.02 ± 0.00[c]	0.01 ± 0.00[d]	0.01 ± 0.01[d]	0.04 ± 0.01[e]
Conductivity (µS/cm)	Min	132.50	56.00	22.70	16.40	29.60	92.90
	Max	144.80	71.10	46.20	17.30	38.00	111.00
	Mean ± SD	139.50 ± 1.29[a]	65.60 ± 4.77[b]	41.56 ± 7.49[c]	16.40 ± 0.28[d]	33.57 ± 3.26[e]	97.01 ± 6.07[f]
Depth (cm)	Min	25.80	17.10	10.20	14.80	10.90	18.20
	Max	48.70	55.00	37.00	49.90	81.50	40.50
	Mean ± SD	36.90 ± 11.47[a]	33.67 ± 14.14[a]	24.59 ± 10.12[a]	30.17 ± 12.04[a]	40.82 ± 28.78[a]	25.58 ± 9.58[a]
Turbidity (NTU)	Min	21.00	0.00	0.00	2.00	42.00	37.00
	Max	22.00	3.00	1.00	8.00	68.00	46.00
	Mean ± SD	21.20 ± 0.45[a]	1.44 ± 0.88[b]	0.33 ± 0.50[b]	4.56 ± 2.55[c]	48.78 ± 17.77[d]	41.50 ± 3.02[ad]
Phosphate (mg/L)	Min	0.83	0.12	0.20	0.11	0.28	0.94
	Max	1.48	0.82	1.89	2.49	8.10	2.46
	Mean ± SD	1.07 ± 0.27[ab]	0.39 ± 0.27[a]	0.86 ± 0.58[ab]	1.13 ± 1.02[ab]	3.23 ± 3.81[c]	1.28 ± 0.59[ab]
Nitrate (mg/L)	Min	0.30	3.80	0.20	0.10	0.00	1.70
	Max	1.30	11.60	1.70	8.10	11.0	3.30
	Mean ± SD	0.93 ± 0.55[a]	7.53 ± 2.76[b]	0.88 ± 0.46[a]	2.02 ± 2.52[a]	1.44 ± 3.61[a]	2.58 ± 0.67[ab]
Ammonia-nitrogen (mg/L)	Min	0.13	0.27	0.10	0.09	0.16	0.94
	Max	0.25	0.59	0.64	0.16	0.51	1.08
	Mean ± SD	0.19 ± 0.08[ab]	0.33 ± 0.11[b]	0.35 ± 0.19[b]	0.14 ± 0.02[a]	0.36 ± 0.17[b]	0.99 ± 0.05[c]
Nitrite (mg/L)	Min	0.17	0.03	0.00	0.00	0.01	0.31
	Max	0.20	0.05	0.010	0.01	0.02	1.50
	Mean ± SD	0.18 ± 0.02[a]	0.04 ± 0.01[b]	0.01 ± 0.00[cd]	0.00 ± 0.00[c]	0.01 ± 0.01[bd]	1.00 ± 0.51[e]

Supplementary Material Table S4

Loadings from Principal Components Analysis for water quality parameters from the sampled rivers

	PC1	PC2	PC3
Eigenvalues	2.29	1.186	1.042
Percentage Variance explained	38.294	19.765	17.362
Cumulative percentage variance	38.294	58.058	75.420
Temperature	**0.886**	0.151	-0.133
Dissolved oxygen	**-0.883**	-0.173	-0.212
Phosphate	-0.170	0.136	**0.792**
Nitrate	0.221	-0.142	0.**600**
Ammonia-nitrogen	0.342	**0.854**	0.028
Nitrite	0.033	**0.944**	-0.035

Extraction was based on eigenvalues > 1. Numbers in bold indicate parameters with high loadings.

Supplementary Material Figure S1

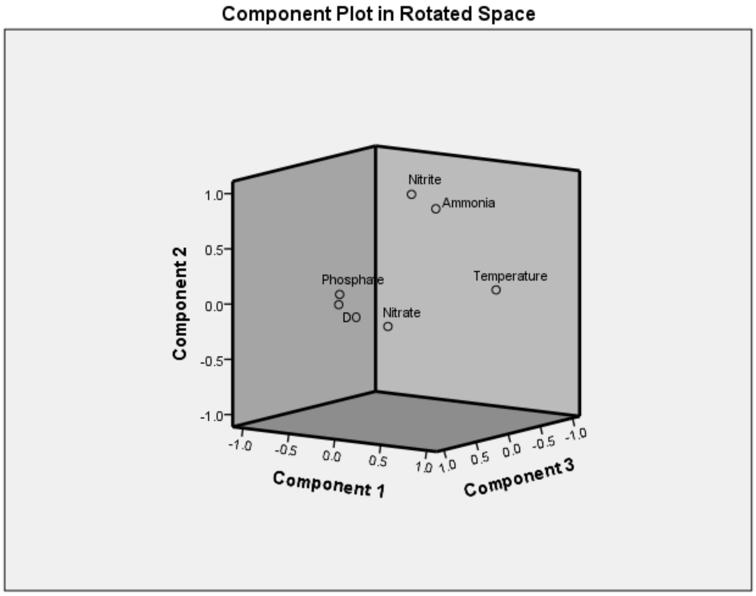

Ordination plot from Principal Components Analysis for water quality parameters from the sampling locations. Rotation Method: Varimax with Kaiser Normalization.

Supplementary Material Table S5

Eigen values and percentage variance of Canonical Correspondence Analysis for water physicochemical parameters from the sampled rivers.

	1	2	3	4	5
Eigenvalue	0.499	0.445	0.199	0.100	0.061
Percentage	38.29	34.14	15.25	7.66	4.65
Cumulative percentage	38.29	72.43	87.68	95.340	99.99
Temperature (℃)	-0.626	-0.140	-0.102	-0.536	-0.698
Dissolved oxygen (mg/L)	0.240	0.364	0.640	0.295	0.804
Phosphate (mg/L)	0.121	0.416	0.052	0.841	-0.578
Nitrate (mg/L)	0.629	-0.156	-0.101	-0.386	-0.002
Ammonia-nitrogen (mg/L)	-0.139	-0.467	0.684	-0.268	-0.340
Nitrite (mg/L)	-0.106	-0.688	0.698	-0.261	-0.128

Supplementary Material Table S6

Eigenvalues and percentage variance of Canonical Correspondence Analysis for anthropogenic factors surrounding the sampled rivers.

	1	2	3	4
Eigenvalue	0.498	0.361	0.199	0.061
Percentage	44.510	32.220	17.820	5.449
Cumulative percentage	44.510	76.730	95.550	99.999
Distance from human settlement	-0.650	0.076	-0.272	0.849
State of accessibility	-0.545	-0.095	-0.849	0.249
Level of protection	0.120	-0.599	-0.141	0.637
Pollution level	-0.397	-0.536	0.457	0.497
Usage for other purposes	0.274	0.049	0.766	-0.789

Supplementary Material Figure S2

Stomach contents of the collected fishes. This file contains some examples of the (A) Insect; (B) Zooplankton; (C) Worm; (D) Fish scale; (E) Detritus; (F) Mud particles; (G) Unicellular algae; (H) Diatoms; (I) Cyanobacteria; (J) Green algae; (K) Red algae; and (L) Plant parts that encountered in the stomachs of fishes. Magnifications range from 4 × to 40 ×.

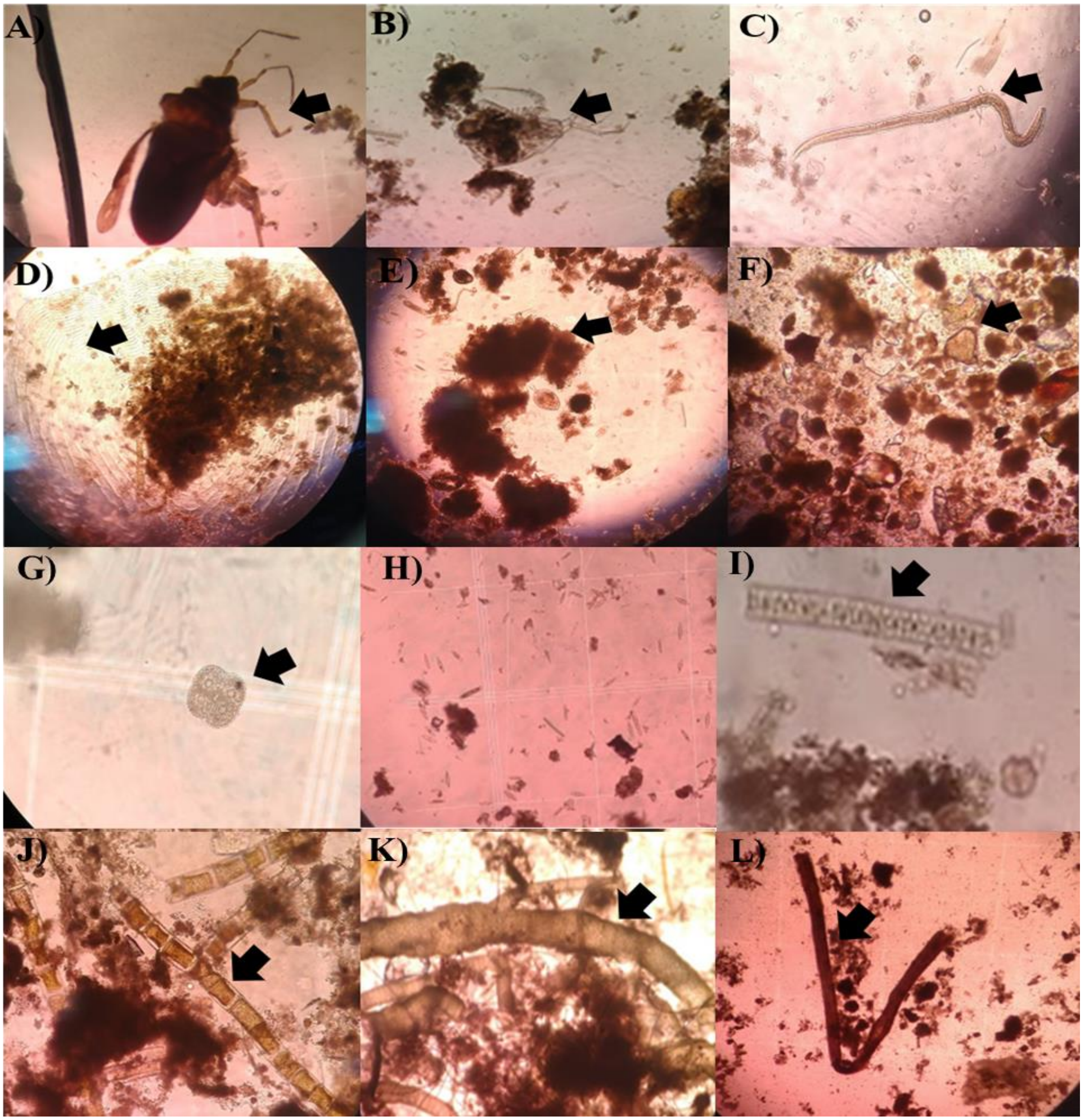

Supplementary Material Table S7

Diet overlaps between fish species from Gombak, Klang, and Langat Rivers based on Morisita-Horn index.

River	Species	C_H index	Interpretation
Gombak	*Mystacoleucus obtusirostris versus Poropuntius normani*	0.98	Significant
	*Mystacoleucus obtusirostris versus Oreochromis niloticus**	0.81	Significant
	*Poropuntius normani versus Oreochromis niloticus**	0.85	Significant
Klang	*Rasbora vulgaris versus Oreochromis niloticus**	0.57	Not significant
Langat	*Mystus singaringan versus Oreochromis niloticus**	0.88	Significant

C_H = Morisita-Horn's diet overlap index. *indicate alien species.

Supplementary Material Table S8

TROPH of native and alien fish species from Gombak, Klang, and Langat Rivers.

River	Species	TROPH ± SE
Gombak	*Mystacoleucus obtusirostris*	2.12 ± 0.15
	Poropuntius normani	2.05 ± 0.11
	*Oreochromis niloticus**	2.06 ± 0.16
Klang	*Rasbora vulgaris*	2.60 ± 0.28
	*Oreochromis niloticus**	2.07 ± 0.16
Langat	*Mystus singaringan*	2.33 ± 0.20
	*Oreochromis niloticus**	2.26 ± 0.20

*indicate alien species.
